# Socio-cultural attitudes toward the body as a predictor of motivation for physical activity in young people brought up in Asian and European culture—Chinese-Polish comparison

**DOI:** 10.1186/s13102-023-00662-y

**Published:** 2023-04-04

**Authors:** Shuai Guo, Bernadetta Izydorczyk, Małgorzata Lipowska, Agata Kamionka, Sebastian Lizińczyk, Urszula Sajewicz-Radtke, Bartosz M. Radtke, Taofeng Liu, Mariusz Lipowski

**Affiliations:** 1 Faculty of Sport and Leisure, Guangdong Ocean University, Zhanjiang, China; 2 Department of Psychology, Gdańsk University of Physical Education and Sport, Gdańsk, Poland; 3 Institute of Psychology, Jagiellonian University, ul. Ingardena 6, Krakow, 30-060 Poland; 4 Institute of Psychology, University of Gdańsk, Gdańsk, Poland; 5 SWPS University of Social Sciences and Humanities, Katowice, Poland; 6 Laboratory of Psychological and Educational Tests, Gdańsk, Poland; 7 School of Physical Education, Zhengzhou University, Zhengzhou, China; 8 Faculty of Social and Humanities, WSB University in Gdańsk, Gdańsk, Poland

**Keywords:** Motivation, Physical activity objectives, Sociocultural standards, Body, Physical appearance, Mass media, Cross-cultural

## Abstract

The influence of sociocultural attitudes toward the body on young people’s physical activity has received increasing attention. However, there is a lack of cross-cultural research in this area. The main aim of this research was to identify the similarities and differences in the sociocultural attitudes toward the body of Polish and Chinese young people who grew up in European and Asian cultures and to analyze their effect on the motivation for physical activity. A cross-sectional research study was conducted among 18- to 30-year-old Polish (n = 259) and Chinese (n = 208) young people. The variables were measured using the Sociocultural Attitudes towards Appearance Questionnaire 3 (SATAQ 3) and the Inventory of Physical Activity Objectives (IPAO). Descriptive and comparative statistics, Spearman’s rho, and the stepwise multiple regression analysis were used. The main analysis showed There are both similarities and significant differences in the performance of young Polish and Chinese men and women on the variables studied; Internalization-Athlete, Pressures, and Internalization-General are universal sociocultural predictors of motivation for physical activity among young people in Poland and China; Information is a specific sociocultural predictor of motivation for physical activity in Polish young people. The cultural nuances need to be considered in understanding young people’s Motivation for undertaking physical activity.

## Introduction

Studies on body image from different regions, such as Europe, Asia and the Americas, generally suggest that sociocultural factors are an important source of desirable body image for young people [1–11]. Social comparisons and attention to cultural norms motivate young people, including those in China and Poland, to take action for an ideal body image, and changing attitudes and behaviors towards physical activity are one of the results [8, 9, 12, 13]. Global public health data confirm that physical inactivity is still highly prevalent among young people in European countries [14], including Poland [15], the Americas [16, 17], and Asia [18], including China [19, 20]. This phenomenon is an essential source of obesity, cardiovascular disease and other health problems in young people [21]. Therefore, investigating the relationship between sociocultural factors and physical activity among young people, and identifying the facilitators and barriers, is a highly significant research topic. Research on physical activity has demonstrated that specific motivation drive engagement in any physical activity [22]. However, the motivation for physical activity vary systematically across demographic variables, such as age [23, 24], gender [25], cultural background [26, 27], and specific preferences [28, 29]. Conducting cross-cultural comparisons of same-gendered young people who grew up in different cultural backgrounds, exploring sociocultural attitudes toward the body, and analyzing the relationship with physical activity motivation can enhance an overall understanding of young people’s physical activity. However, there are relatively few such studies available. Therefore, the main aim of this research was to identify the similarities and differences in the sociocultural attitudes toward the body of Polish and Chinese young people who grew up in European and Asian cultures and to analyze their effect on the motivation for physical activity.

This relationship can be considered based on self-determination theory and social cognitive theory. Self-determination theory is a macro-theory of human motivation concerned with how social contextual factors support or hinder people’s thriving by satisfying their basic psychological needs for competence, relatedness, and autonomy [30, 31]. Research on physical activity from the self-determination theory perspective has demonstrated that motivation for physical activity is related to body satisfaction, disordered eating behaviors, and social-cultural attitudes about the body [32–34]. Thompson and his team [35] proposed a three-factor model of sociocultural, psychological, and eating behavior based on sociocultural theory. The model argues that individuals are pressured by powerful social factors (i.e., media, information) to comply with culturally defined appearance ideals and that the internalization of these ideals will alter an individual’s behavior to meet social norms [36]. Anić et al. [34] surveyed young women aged 18–29, and the results showed that sociocultural pressure and internalization of appearance ideals mediate BMI and exercise (or physical activity) motivation in women. Jankauskiene and team [1] tested the association of sociocultural ideas of appearance with health-compromising physical activity in a study of a sample of 736 (437 women, 299 men) adolescents, body image concerns and internalization of sociocultural norms were significantly higher in the health-compromising physical activity group. Hu et al. [37] conducted a systematic review from the social-ecological model perspective and showed that parental, friend, and teacher support was a positive predictor of adolescent participation in physical activity. The literature review confirms that sociocultural attitudes toward the body are closely related to motivation for physical activity in young people. However, social culture is closely related to historical traditions, environment, ethnicity, etc., and will evolve with the development of society. Therefore, considering the potential differences and similarities in sociocultural, psychological, and other aspects of young men and women growing up in different environments and ethnic groups, it is necessary to conduct cross-cultural comparisons of the sociocultural attitudes toward the body of young people growing up in different cultures and measure its significance for motivation for physical activity. For evidence-based practice, measuring motivation for physical activity in young people requires constant verification with various European, Asian, and American populations to expand knowledge on universal and specific sociocultural predictors of motivation for physical activity [38].

With globalization and media information development, the aesthetic standards of different countries’ social cultures are merging and unifying [39]. Still, the researchers confirmed cross-cultural differences, such as the desire to be thin among young people in Asian countries, China [40]; and in the Americas, young people in the United States are very concerned about muscles [39]. A literature review of the sociocultural attitudes toward the body influencing motivation for physical activity in young people confirms that further research is needed in different ethnic groups to determine which factors are universal and which are specific. Culture shapes the context in which people’s ideas about their bodies are formed, and there are many classifications and typologies of culture, such as the dichotomy of individualistic and collectivistic cultures, where individualistic types focus on the importance of their own needs, self, and individual characteristics; collectivistic consider the importance of group perspectives, needs, and goals more critical [41, 42]. The Polish cultural identity is individualistic; on the contrary, the Chinese cultural identity is collectivistic [43]. Although the cultural factors that influence the growth of young people in Poland and China may be different, there are some cultural similarities due to the increasing internalization of the sociocultural norms of globalization promoted by mass media in both countries [44].

Drawing on the research of Thompson et al. [35, 36] and Fan et al. [45], we surveyed young people in Poland and China with SATAQ-3 to assess the explanatory variables.

## Research variables and research questions

The independent variable was the sociocultural attitude towards the body. Based on the literature review [35, 36], this variable is defined as a variable with a four-factor structure and describes the degree of internalization of sociocultural standards of body appearance. The first component was the internalization of sociocultural standards, which describes the level of internalization of sociocultural standards of body and appearance promoted by the mass media (TV, magazines, commercials, etc.). The second component was pressure from sociocultural standards, which describes the level of pressure a person feels about the sociocultural standards of body and appearance promoted by the mass media. The third component was information, which describes the frequency with which a person searches for information on sociocultural standards of body and appearance promoted by the mass media. The fourth component was the internalization of the athlete, which describes the level of internalization of the athletic body ideas promoted by the mass media.

The dependent variable was the motivation for physical activity. The physical activities here are performed without medical recommendations. Drawing on the study of Lipowski et al. [22], this variable describes the objectives of consciously undertaken physical activity by the person and the motivational function of the objectives.

The following research questions were considered:

1. Are there differences in motivations for physical activity and sociocultural attitudes toward the body among Polish and Chinese same-sex young people brought up in European and Asian cultures? If so, which characteristics are common to them and which are specific?
2. How do sociocultural attitudes toward the body among young people in Poland and China relate to their objectives of physical activity and the motivational effects of these objectives? If so, are there cross-cultural differences in this relationship?

## Materials and methods

### Participants

The groups were selected by purposeful sampling. The inclusion criteria were: age (18–30 years old), Polish or Chinese nationality and growing up in that country (lived with the family from childhood to now in Poland or China for the respective groups), lack of physical disability or somatic diseases that prevents physical activity, students or graduates in the humanities and social sciences. The criteria were validated through a questionnaire, which allowed the identification of exclusion factors.

The research was carried out simultaneously in 2021 in two academic cities in Poland (Krakow and Gdansk) and two academic cities in China (Beijing and Zhengzhou). Qualified researchers with psychological training (students and team members of the study authors) conducted the research. The researchers first disseminated information about the project among students majoring in humanities and social sciences at the aforementioned urban university and collected the email addresses of voluntary participants who met the selection criteria. Simultaneously, these students were asked to invite their peers to participate and collect their email addresses. Finally, the project details and a link to the online survey were emailed to participants. Participants must first complete an electronic informed consent form in order f access the questionnaire completion page.

From January to December 2021, we distributed research materials to 800 individuals, comprising 400 participants from Poland and 400 from China. Ultimately, 594 individuals (303 from Poland and 291 from China) participated in the study, resulting in 76% and 73% response rates for Poland and China, respectively. After preliminary screening to exclude samples which did not meet the inclusion criteria, provided conflicting information, or exhibited response biases, 44 young Polish and 83 young Chinese participants were excluded from the analysis. The final sample consisted of 186 Polish females (Mean age = 25.4, SD = 3.03), 73 Polish males (Mean age = 24.3, SD = 3.37), 98 Chinese females (Mean age = 22.2, SD = 3.11), and 110 Chinese males (Mean age = 22.3, SD = 2.82). Among the Chinese sample, 75.5% of females and 74.5% of males were university students, while 52.2% of females and 60.3% of males were university students in the Polish sample. The mean BMI for young Polish females and males was 21.32 and 24.16, respectively, while the mean BMI for young Chinese females and males was 21.67 and 23.27, respectively. All participants had humanities and social sciences backgrounds and had no prior experience as athletes or professional sports learners.

### Procedure

The data used for this study were part of a large international research project registered in the Protocol Registration and Results System (ClinicalTrials.gov; https://clinicaltrials.gov/ct2/show/NCT04432038). The procedure carried out in the project consisted of an online survey. The study was carried out in accordance with the Code of Ethics of the World Medical Association (Declaration of Helsinki) for research involving humans. The protocol of this study was approved by the Ethics Board for Research Projects at the Institute of Psychology, University of Gdansk, Poland (decision no. 33/2020). All participants were acquainted with the purpose of the conducted research and asked to complete an electronic informed consent form before registration on the project’s website.

### Methods

#### The sociocultural attitudes towards appearance questionnaire 3 (SATAQ 3)

The independent variables were measured by the Polish [46] and Chinese [47] versions of the Sociocultural Attitudes Towards Appearance Questionnaire 3 (SATAQ 3) [36]. The SATAQ 3 has a total of 30 items, organized into four-factor structures, which contain the following subscales: Internalization-General (assess the extent to which respondents internalization of sociocultural standards; consist of nine items; e.g., I would like my body to look like the people who are in the movies), Internalization-Athlete (measure to what extent respondents internalized athletic body standards; consist of five items; e.g., I wish I looked as athletic as sports stars), Pressures (assess the level of pressure of sociocultural standards felt by a person; consist of seven items; e.g., I have felt pressure from TV and magazines to be thin), Information (measure the frequency of seeking for information on sociocultural standards of body and appearance; consist of nine items; e.g., Magazine advertisements are an important source of information about fashion and ‘‘being attractive’’). The SATAQ 3 questionnaire was completed by the participants on a 5-point Likert scale. The Polish and Chinese versions of the SATAQ 3 demonstrated sound psychometric properties, including internal consistency and convergent validity [46, 47]. In the present study, the Cronbach’s alpha coefficients were: Internalization-General (0.897 in Polish studies and 0.868 in Chinese studies), Internalization-Athlete (0.859 in Polish studies and 0.654 in Chinese studies), Pressures (0.950 in Polish studies and 0.857 in Chinese studies), Information (0.862 in Polish studies and 0.793 in Chinese studies).

#### The inventory of physical activity objectives (IPAO)

We measured the dependent variable using the Inventory of Physical Activity Objectives (IPAO) of Polish scholars Lipowski and Zaleski [48]. There are some extensive demographic questions in IPAO, such as gender, age, height, weight, engagement in physical activity, etc. IPAO also includes two scales that measure the objectives of undertaken physical activity (consisting of twelve items, on a 5-point Likert scale) and the motivational function of the objectives (consisting of eighteen items, on a 5-point Likert scale). On the scale of the objectives of undertaken physical activity, the attitude of the subjects toward particular objectives was measured (e.g., Physical fitness, being ‘in shape’). Four subscales of goal-oriented behaviors associated with physical activity are distinguished in the scale of the motivational function of the objectives: motivational value (e.g., I am deeply convinced that I will achieve this goal), time management (e.g., I devote my entire free time to accomplishing this goal), persistence in action (e.g., I am worried that I won’t fully achieve this goal), and motivational conflict (e.g., I have other goals that are just as satisfying as the one described). The Chinese version of the questionnaire was translated using a standard forward-backwards translation procedure. IPAO has shown sound psychometric properties in existing studies of in Poland and China [48, 49]. In the present study, Cronbach’s alpha for the scale of the motivational function of objectives of IPAO was: Polish version = 0.831, Chinese version = 0.899.

### Statistical methods

According to the research objectives and research question, statistical analyses were performed in Excel (Microsoft office 365) and IBM SPSS Statistics 28.0.

Stage 1 - descriptive statistics for all variables, include mean, median, standard deviation, variance, skewness, kurtosis, and percentile.

Stage 2 - as the subject variables were not normally distributed, the Mann-Whitney U test was used to measure the significance of the difference between the two groups.

Stage 3 - the significance of the difference in the strength of the relationship and the strength of the correlation between the two groups of variables was measured using Spearman’s rank correlation coefficient.

Stage 4 - measure the strength of the relationship between the dependent and independent variables using stepwise multiple regression analysis. This stage aimed to search for predictors of the dependent variables in the groups of Polish and Chinese for young men and young women.

First, we conducted separate tests to assess the multicollinearity among the four independent variables in Poland and China’s male and female samples. Table 1 shows that all tolerance (TOL) values are greater than 0.10, and all variance inflation factor (VIF) values are less than 10, indicating no multicollinearity among the independent variables. Second, we performed stepwise multiple regression analysis to determine the most predictive independent variables for the dependent variable in Poland and China’s male and female samples. Taking the “Health” variable in the female sample of Poland as an example, we selected the independent and dependent variables and then used the stepwise regression analysis method. The criterion for selecting variables was based on the F-probability value, with a critical value of 0.05 for inclusion in the model and 0.10 for exclusion. Both forward selection and backward elimination methods were employed, and the best multiple regression analysis models were selected using statistical software features. We followed the same steps to test the most predictive independent variables for each dependent variable (Health; Physical Fitness; Company of Others; Fit, Shapely Body; Well-Being; Fashion; Boosting Confidence; Pleasure from Physical Activity; Escape from Everyday Life; Managing Stress; Fulfilling the Need for Activity; Promoting Physical Activity; Motivational Value; Time-Management; Persistence in Action; Motivational Conflict) in Poland and China’s male and female samples.

**Table 1:** Results of multicollinearity test for all independent variables

Independent variable	Polish				Chinese			
	Female		Male		Female		Male	
	TOL	VIF	TOL	VIF	TOL	VIF	TOL	VIF
Internalization-General	0.368	2.720	0.286	3.496	0.346	2.888	0.554	1.805
Information	0.307	3.260	0.324	3.088	0.638	1.567	0.649	1.542
Pressures	0.287	3.479	0.487	2.054	0.359	2.786	0.497	2.014
Internalization-Athlete	0.741	1.350	0.618	1.619	0.799	1.252	0.789	1.267

*Note: TOL, tolerance value; VIF, variance inflation factor value*

## Results

### Characteristics of objectives of undertaken physical activity, the motivational function of the objectives, and sociocultural attitude of body appearance in young Polish and Chinese (differences between the groups)

A comparative analysis of all variables in the Polish and Chinese groups showed that (Table 2):

1. The most important objective for young Polish and Chinese men and women undertaking physical activity is Well-Being. Among young Polish and Chinese women, the 12 physical activity objectives showed significant differences only in the importance of the Company of Others objective and the Managing Stress objective, with young Polish women performing better on the Company of Others objective and young Chinese women performing better on the Managing Stress objective. The importance of more physical activity objectives among young Polish and Chinese men showed significant differences, with young Polish men performing better in terms of the importance of the Escape from Everyday Life objective, while young Chinese men performed better in terms of the importance of Health objective, Fit, Shapely Body objective, Fashion objective, Boosting Confidence objective and Promoting Physical Activity objective.
2. In terms of motivational functions of objectives, Polish and Chinese young women showed significant differences in Motivational Value, Persistence in Action, and Motivational Conflict, with Polish women having a higher of Motivational Value than Chinese women and a lower of Persistence in Action, and Motivational Conflict than Chinese women. On the other hand, Polish and Chinese young men showed significant differences in Time-Management, Persistence in Action, and Motivational Conflict, and all three variables were higher for Chinese young men than for Polish young men.
3. In terms of sociocultural attitudes toward the body, Polish and Chinese young women showed significant differences in the variables Information and Internalization-Athlete, with young Polish women showing significantly lower levels on the Information variable than Chinese young women and higher levels on the Internalization-Athlete variable than Chinese young women. Polish and Chinese young men showed significant differences in the three variables of Internalization-General, Information, and Pressures, with Chinese young men showing significantly higher on all three variables than Polish young men.

**Table 2:** Comparative analysis between groups of Polish and Chinese young people in terms of the variables included in the study model

Variables	Female						Male					
	Polish (n = 186)		Chinese (n = 98)		Differences		Polish (n = 73)		Chinese (n = 110)		Differences	
	M	SD	M	SD	U	p	M	SD	M	SD	U	p
Health	4.60	0.70	4.53	0.78	9351.00	0.658	4.08	1.00	4.40	0.92	3173.00	0.008
Physical Fitness	4.60	0.67	4.52	0.75	9505.50	0.471	4.45	0.90	4.34	1.03	4145.00	0.663
Company of Others	3.87	1.14	3.42	1.29	10909.00	0.005	3.73	1.26	3.99	1.09	3585.50	0.199
Fit, Shapely Body	4.58	0.71	4.39	0.90	10098.00	0.074	4.04	1.06	4.42	0.88	3169.00	0.008
Well-Being	4.77	0.48	4.59	0.80	9802.50	0.151	4.53	0.78	4.54	0.80	3995.00	0.944
Fashion	3.41	1.32	3.16	1.24	10239.50	0.079	2.41	1.45	3.47	1.26	2347.50	< 0.001
Boosting Confidence	3.62	1.29	3.83	1.08	8477.50	0.316	2.81	1.30	4.07	1.00	1875.00	< 0.001
Pleasure from Physical Activity	4.55	0.67	4.37	0.89	9910.50	0.158	4.52	0.69	4.39	0.80	4289.00	0.373
Escape from Everyday Life	3.67	1.05	3.54	1.16	9578.50	0.464	3.97	1.08	3.54	1.16	4909.00	0.008
Managing Stress	4.04	0.88	4.26	0.90	7741.00	0.026	3.99	1.25	4.11	1.04	3938.50	0.815
Fulfilling the Need for Activity	4.28	0.79	4.12	1.01	9637.00	0.390	4.12	1.07	4.19	0.92	3965.00	0.878
Promoting Physical Activity	3.74	1.15	3.74	1.09	9169.00	0.931	3.07	1.43	3.67	1.10	3013.50	0.003
Motivational Value	33.06	5.19	29.73	4.85	12574.00	< 0.001	32.33	5.32	31.38	4.80	4388.50	0.286
Time-Management	16.98	4.46	16.53	3.64	9997.00	0.178	14.70	4.39	17.78	3.41	2258.50	< 0.001
Persistence in Action	7.78	2.67	9.76	2.73	5468.50	< 0.001	7.62	2.64	10.32	2.66	1926.00	< 0.001
Motivational Conflict	7.10	1.84	7.87	1.63	7044.50	0.001	7.26	1.95	8.01	1.71	3140.50	0.011
Internalization-General	24.98	8.15	26.83	6.83	8169.50	0.151	19.07	7.16	27.51	4.96	1272.50	< 0.001
Information	24.09	7.88	27.73	5.32	6846.00	< 0.001	16.68	6.37	27.28	4.01	663.00	< 0.001
Pressures	20.01	7.94	19.65	5.17	9873.50	0.248	12.04	5.84	19.25	4.23	1324.00	< 0.001
Internalization-Athlete	17.93	5.07	15.43	3.21	12182.00	< 0.001	15.81	4.62	15.75	2.59	4276.50	0.453

*Note: The significance threshold was set at 0.05; N, number of people; M, mean; SD, standard deviation; U, Mann-Whitney U; p, significance level*

### The relation between studied variables among Polish and Chinese young men and women

The results of correlation obtained for young men and women in the Poland group are listed in Table 3. In the young Polish women, the frequency of seeking information about body image and physical appearance from mass media, the endorsement, and acceptance of an athletic body ideal were significant correlations with most the physical activity objectives, the motivational function of the objectives, these correlations are both positive and negative. In Polish young men, the frequency of seeking information about body image and physical appearance from mass media, the endorsement, and acceptance of an athletic body ideal was still significant correlations with half of the physical activity objectives, which are all positive.

**Table 3:** Correlation analysis for all variables for the group of young Polish men and women (Spearman’s rho coefficient)

IPAO	SATAQ 3							
	Internalization-General		Information		Pressures		Internalization-Athlete	
	Female	Male	Female	Male	Female	Male	Female	Male
Health	0.13	0.18	0.26^**^	0.25^*^	0.22^**^	0.15	0.32^**^	0.05
Physical Fitness	-0.13	0.10	-0.12	0.02	-0.08	-0.20	0.07	0.18
Company of Others	0.03	0.33^**^	0.15^*^	0.25^*^	-0.01	0.07	0.19^**^	0.37^**^
Fit, Shapely Body	0.28^**^	0.17	0.29^**^	0.34^**^	0.28^**^	0.08	0.43^**^	0.29^*^
Well-Being	0.05	0.00	0.05	0.06	0.02	-0.22	0.19^**^	0.24^*^
Fashion	0.41^**^	0.37^**^	0.53^**^	0.52^**^	0.40^**^	0.37^**^	0.47^**^	0.13
Boosting Confidence	0.42^**^	0.19	0.60^**^	0.25^*^	0.48^**^	0.19	0.56^**^	0.26^*^
Pleasure from Physical Activity	-0.10	-0.22	-0.14^*^	-0.22	-0.13	-0.14	0.10	0.10
Escape from Everyday Life	-0.03	0.00	-0.21^**^	0.06	-0.084	-0.13	-0.13	0.25^*^
Managing Stress	-0.06	0.07	-0.32^**^	0.15	-0.18^*^	-0.05	-0.24^**^	0.20
Fulfilling the Need for Activity	-0.10	0.05	-0.11	0.15	-0.09	-0.09	0.09	0.14
Promoting Physical Activity	-0.01	0.37^**^	0.02	0.30^**^	0.00	0.20	0.22^**^	0.35^**^
Motivational Value	-0.02	-0.04	0.14	-0.06	0.06	-0.26^*^	0.39^**^	0.30^*^
Time-Management	0.11	0.15	0.42^**^	0.21	0.24^**^	0.00	0.60^**^	0.26^*^
Persistence in Action	0.06	0.21	-0.23^**^	0.20	-0.16^*^	0.20	-0.42^**^	0.03
Motivational Conflict	-0.29^**^	-0.07	-0.25^**^	-0.14	-0.17^*^	-0.18	-0.13	-0.02

*Note: SATAQ 3- the Sociocultural Attitudes Towards Appearance Questionnaire 3; IPAO- the Inventory of Physical Activity Objectives;* ^*^ *P < 0.05;* ^**^ *P < 0.01*

The results obtained for young men and women in the Chinese group are listed in Table 4. In Chinese young women, the endorsement and acceptance of an athletic body ideal were the significant correlations with most physical activity objectives; these correlations are both positive and negative. In Chinese young men, the sociocultural attitude toward the body and motivational function of the physical activity objectives did not significant correlation.

**Table 4:** Correlation analysis for all variables for the group of young Chinese men and women (Spearman’s rho coefficient)

IPAO	SATAQ 3							
	Internalization-General		Information		Pressures		Internalization-Athlete	
	Female	Male	Female	Male	Female	Male	Female	Male
Health	0.07	0.08	0.10	-0.06	0.07	0.01	0.22^*^	0.06
Physical Fitness	-0.06	-0.16	-0.04	-0.29^**^	-0.14	-0.26^**^	0.21^*^	0.06
Company of Others	0.07	-0.12	0.10	0.06	0.13	0.03	0.11	0.05
Fit, Shapely Body	0.14	0.00	0.19	-0.08	0.14	-0.04	0.19	0.02
Well-Being	0.00	-0.17	-0.02	-0.06	0.04	-0.15	0.14	-0.01
Fashion	0.17	-0.07	0.04	0.20^*^	0.12	0.15	0.06	-0.15
Boosting Confidence	0.24^*^	0.02	0.18	0.15	0.25^*^	-0.01	0.25^*^	0.10
Pleasure from Physical Activity	0.01	-0.18	0.12	-0.12	0.01	-0.23^*^	0.22^*^	0.07
Escape from Everyday Life	0.29^**^	-0.23^*^	0.20^*^	-0.02	0.32^**^	-0.12	0.30^**^	-0.15
Managing Stress	0.03	-0.24^*^	0.12	-0.02	0.08	-0.16	0.10	-0.20^*^
Fulfilling the Need for Activity	0.06	-0.09	0.07	0.16	-0.02	-0.01	0.28^**^	0.11
Promoting Physical Activity	-0.07	-0.06	-0.01	0.17	-0.09	0.11	0.13	-0.11
Motivational Value	0.08	-0.04	0.15	0.01	0.04	0.04	0.10	0.09
Time-Management	0.15	-0.15	0.13	0.05	0.20	0.01	0.11	0.04
Persistence in Action	0.37^**^	-0.02	0.34^**^	0.13	0.35^**^	0.17	0.13	0.00
Motivational Conflict	0.00	-0.13	0.10	-0.08	-0.05	-0.04	0.04	0.03

*Note: SATAQ 3- the Sociocultural Attitudes Towards Appearance Questionnaire 3; IPAO- the Inventory of Physical Activity Objectives;* ^*^ *P < 0.05;* ^**^ *P < 0.01*

### The sociocultural predictors of motivation for physical activity in the Polish and Chinese young people

Table 5 shows the stepwise regression analysis results for the Polish and Chinese young men and women groups. It is worth mentioning that some of the dependent variables in the Chinese and Polish samples of men and women did not find relevant predictor variables and could not be executed in stepwise regression analysis. It can be seen from Table 5 that:

1. Internalization-Athlete was a common predictor factor for the importance of physical activity objectives of Health, Fit, Shapely Body, Well-Being, Boosting Confidence, and Pleasure from Physical Activity in the Polish and Chinese young women. Their β coefficients are all positive, indicating that the effects of Internalization-Athlete on these objectives are all positive.
2. Pressures were a common predictor factor for the importance of physical activity objectives of Physical Fitness and Pleasure from Physical Activity in the Polish and Chinese young men. Their β coefficients are all negative, indicating that the effects of Pressures on both objectives are all negative.
3. Among the predictors of physical activity objectives, Information was a common predictor factor for the importance of physical activity objectives of Fashion in Polish young women and men, Internalization-Athlete was a common predictor factor for the importance of physical activity objectives of Boosting Confidence and Promoting Physical Activity in the Polish young women and men, and their effects are all positive. In addition, the predictors of physical activity objectives showed significant differences in Polish women and men. Such as Pressures is a predictor of Health objective in young Polish women, but no predictor effect in young Polish men; Information is a predictor of Well-Being objective in young Polish men, but no predictor effect in young Polish women; etc. At the same time, the predictors of physical activity objectives were more significantly different among young Chinese men and women, who did not have any common predictors.
4. Internalization-Athlete was a common predictor factor for the development of Motivational Value and Time-Management among Polish young men and women, and their effects are all positive. Apart from that, the predictors of the motivational function of objectives showed significant differences in Polish and Chinese women and men. Such as, Pressure is a positive predictor of Persistence in Action in Chinese young women but has no predictor effect in Chinese young men; conversely. Internalization-General is a negative predictor of Motivational Conflict in young Polish women but does not affect young Polish men.

**Table 5:** Comparison of predictor factors for the development of motivation for physical activity in the Polish and Chinese young men and women

Dependent variable	Polish		Chinese	
	Female	Male	Female	Male
Health	R^2^ = 0.092 F = 9.259^***^ **M1**, Adj R^2^ = 0.066 **M2**, Adj R^2^ = 0.082 Predictors: **IA**, β = 0.202^**^ **P**, β = 0.157^*^	R^2^ = 0.063 F = 4.781^*^ Adj R^2^ = 0.050 Predictors: **I**, β = 0.251^*^	R^2^ = 0.063 F = 6.453^*^ Adj R^2^ = 0.053 Predictors: **IA**, β = 0.251^*^	
Physical Fitness		R^2^ = 0.139 F = 5.632^**^ **M1**, Adj R^2^ = 0.062 **M2**, Adj R^2^ = 0.114 Predictors: **P**, β=-0.462^***^ **IG**, β = 0.315^*^		R^2^ = 0.046 F = 5.216^*^ AdjR^2^ = 0.037 Predictors: **P**, β=-0.215^*^
Company of Others	R^2^ = 0.045 F = 8.645^**^ Adj R^2^ = 0.040 Predictors: **IA**, β = 0.212^**^	R^2^ = 0.115 F = 9.217^**^ Adj R^2^ = 0.102 Predictors: **IG**, β = 0.339^**^		
Fit, Shapely Body	R^2^ = 0.188 F = 21.208^***^ **M1**, Adj R^2^ = 0.157 **M2**, Adj R^2^ = 0.179 Predictors: **IA**, β = 0.338^***^ **IG**, β = 0.176^*^	R^2^ = 0.152 F = 6.277^**^ **M1**, Adj R^2^ = 0.081 **M2**, Adj R^2^ = 0.128 Predictors: **I**, β = 0.535^***^ **P**, β=-0.333^*^	R^2^ = 0.084 F = 8.812^**^ Adj R^2^ = 0.075 Predictors: **IA**, β = 0.290^**^	
Well-Being	R^2^ = 0.030 F = 5.628^*^ Adj R^2^ = 0.024 Predictors: **IA**, β = 0.172^*^	R^2^ = 0.233 F = 10.642^***^ **M1**, Adj R^2^ = 0.092 **M2**, Adj R^2^ = 0.211 Predictors: **P**, β=-0.664^***^ **I**, β = 0.494^***^	R^2^ = 0.102 F = 5.422^**^ **M1**, Adj R^2^ = 0.039; **M2**, Adj R^2^ = 0.084 Predictors: **IA**, β = 0.329^**^ **IG**, β=-0.255^*^	
Fashion	R^2^ = 0.336 F = 46.290^***^ **M1**, Adj R^2^ = 0.258 **M2**, Adj R^2^ = 0.329 Predictors: **I**, β = 0.351^***^ **IA**, β = 0.316^***^	R^2^ = 0.245 F = 22.989^***^ Adj R^2^ = 0.234 Predictors: **I**, β = 0.495^***^		
Boosting Confidence	R^2^ = 0.397 F = 60.120^***^ **M1**, Adj R^2^ = 0.334 **M2**, Adj R^2^ = 0.390 Predictors: **I**, β = 0.437^***^ **IA**, β = 0.283^***^	R^2^ = 0.093 F = 7.244^**^ Adj R^2^ = 0.080 Predictors: **IA**, β = 0.304^**^	R^2^ = 0.071 F = 7.374^**^ Adj R^2^ = 0.062 Predictors: **IA**, β = 0.267^**^	
Pleasure from Physical Activity	R^2^ = 0.066 F = 6.477^**^ **M1**, Adj R^2^ = 0.017 **M2**, Adj R^2^ = 0.056 Predictors: **I**, β=-0.272^***^ **IA**, β = 0.244^**^	R^2^ = 0.059 F = 4.473^*^ Adj R^2^ = 0.046 Predictors: **P**, β=-0.243^*^	R^2^ = 0.076 F = 7.920^**^ Adj R^2^ = 0.067 Predictors: **IA**, β = 0.276^**^	R^2^ = 0.040 F = 4.497^*^ AdjR^2^ = 0.031 Predictors: **P**, β=-0.200^*^
Escape from Everyday Life	R^2^ = 0.065 F = 6.369^**^ **M1**, Adj R^2^ = 0.028 **M2**, Adj R^2^ = 0.055 Predictors: **I**, β=-0.370^***^ **IG**, β = 0.260^*^	R^2^ = 0.065 F = 4.946^*^ Adj R^2^ = 0.052 Predictors: **IA**, β = 0.255^*^	R^2^ = 0.131 F = 7.140^**^ **M1**, Adj R^2^ = 0.074; **M2**, Adj R^2^ = 0.12 Predictors: **P**, β = 0.232^*^ **IA**, β = 0.225^*^	R^2^ = 0.038 F = 4.218^*^ AdjR^2^ = 0.029 Predictors: **IG**, β=-0.194^*^
Managing Stress	R^2^ = 0.136 F = 14.392^***^ **M1**, Adj R^2^ = 0.087 **M2**, Adj R^2^ = 0.126 Predictors: **I**, β=-0.524^***^ **IG**, β = 0.305^**^			R^2^ = 0.080 F = 9.377^**^ AdjR^2^ = 0.071 Predictors: **IG**, β=-0.283^**^
Fulfilling the Need for Activity			R^2^ = 0.103 F = 11.027^**^ Adj R^2^ = 0.94 Predictors: **IA**, β = 0.321^**^	
Promoting Physical Activity	R^2^ = 0.074 F = 14.634^***^ Adj R^2^ = 0.069 Predictors: **IA**, β = 0.271^***^	R^2^ = 0.105 F = 8.354^**^ Adj R^2^ = 0.069 Predictors: **IA**, β = 0.324^**^		
Motivational Value	R^2^ = 0.153 F = 16.469^***^ **M1**, Adj R^2^ = 0.112 **M2**, Adj R^2^ = 0.143 Predictors: **IA**, β = 0.415^***^ **IG**, β=-0.201^**^	R^2^ = 0.182 F = 7.808^***^ **M1**, Adj R^2^ = 0.080 **M2**, Adj R^2^ = 0.159 Predictors: **P**, β=-0.355^**^ **IA**, β = 0.303^**^		
Time-Management	R^2^ = 0.403 F = 40.989^***^ **M1**, Adj R^2^ = 0.295 **M2**, Adj R^2^ = 0.310 **M3**, Adj R^2^ = 0.393 Predictors: **IA**, β = 0.468^***^; **IG**, β= -0.433^***^; **I**, β = 0.460^***^	R^2^ = 0.056 F = 4.222^*^ Adj R^2^ = 0.043 Predictors: **IA**, β = 0.237^*^		
Persistence in Action	R^2^ = 0.266 F = 22.014^***^ **M1**, Adj R^2^ = 0.295 **M2**, Adj R^2^ = 0.310 **M3**, Adj R^2^ = 0.393 Predictors: **IA**, β=-0.398^***^; **IG**, β = 0.553^***^; **P**, β= -0.430^***^	R^2^ = 0.091 F = 7.101^**^ Adj R^2^ = 0.043 Predictors: **P**, β = 0.302^**^	R^2^ = 0.182 F = 21.399^***^ Adj R^2^ = 0.174 Predictors: **P**, β = 0.427^***^	
Motivational Conflict	R^2^ = 0.096 F = 19.430^***^ Adj R^2^ = 0.091 Predictors: **IG**, β=-0.309^***^			

*Note: R*^*2*^, coefficient of determination; F, Fisher test;^*^P < 0.05, ^**^P < 0.01, ^***^P < 0.001;***M1***, *model 1;****M2***, *model 2; Adj R*^*2*^, *model-adjusted coefficients of determination;β, standardized regression coefficient;****IG***, *Internalization-General;****I***, *Information;****P***, *Pressures;****IA***, *Internalization-Athlete.*

### Discussion

#### Similarities and differences between Polish and Chinese young people

The results of this study confirm that there are significant differences between Polish and Chinese young people in terms of the importance of physical activity objectives and the motivation function of the objectives, but this difference is only presented in some of the variables. Well-Being objective and Physical Fitness objective show high importance in both Polish and Chinese young men and women. Polish and Chinese young women differed significantly in the importance of only two of the 12 physical activity objectives, Company of Others and Managing Stress, while Polish and Chinese young men differed significantly in six objectives, Health, Fit, Shapely Body, Fashion, Boosting Confidence, Escape from Everyday Life, and Promoting Physical Activity. Research on motivation for physical activity among Chinese young people also demonstrated that male participants pay more attention to physical fitness, shape, and well-being goals [49]; female participants focus more on health, well-being, and fit body [50, 51]. Lipowski et al. [22, 52] indicate, that well-being, physical fitness, and health were the most important objectives of physical activity for young men and women in their research conducted on Polish young people. The study by Kuśnierz et al. [53] also demonstrates this theme. It is worth mentioning that the study by Wilczyńska et al. [49] on the motivation for physical activity among young people in China and Poland was carried out under the influence of the COVID-19 epidemic, which is similar to the social background of the questionnaire survey conducted in this study in 2021, and they obtained the same understanding with this study.

Compared to young Chinese men and women, the physical activity objectives of young Polish men and women show greater efficiency and persistence of action, and the ability to cope with adversity, and their physical activity objectives are less vulnerable to conflict with other objectives. The physical activity objectives of young Polish women have a greater influence on the actions taken by the individual. Young Chinese men are more focused to planning, arranging, and organizing their physical activity time. Although there are no comparative studies describing the motivational function of physical activity objectives in Polish and Chinese young people, some studies have confirmed that the gender of the participants and whether they have an athlete experience modulates the motivational functions of physical activity objectives [22, 52]. The present study demonstrates that there are both similar and significantly different perceptions of the importance of the purpose of physical activity and the motivational value of the goal between young Polish and Chinese men and women, this can confirm the findings of other authors.

On the other hand, the results of this study also show that there are significant differences between young people in Poland and China in terms of the average level of some variables of sociocultural attitudes towards the body. Young Polish women seek information about body image and appearance from the mass media less frequently than young Chinese women, but they have higher endorsement and acceptance of an athletic body ideal than young Chinese women. Young Chinese men prefer to have the same appearance as people in mass media such as TV or magazines more often than young Polish men, seek information about body image and physical appearance from mass media more frequently, and feel more pressure from mass media regarding physical appearance standards. A comparative study of the body image of university students in 22 countries in regions Pacific Asia, North-Western Europe and the USA, Central and Eastern Europe, et al. by Wardle et al. [54] demonstrated that both men and women from Asian countries showed higher levels of weight concern and weight loss attempts. A cross-cultural study of Polish (age m = 21.5) and Vietnamese (age m = 20.4) young people by Lipowska et al. [55] showed that Polish young women are more satisfied with their body appearance than Vietnamese young women, and they pay more attention to body strength and muscles. A study by Izydorczyk et al. [44] on young Polish and Japanese women showed that young Japanese women were significantly higher than Polish women in terms of seek information about body image and appearance from the mass media. Some studies on sociocultural attitudes towards appearance also show that young Chinese men show higher levels of Internalization-General, Information, and Pressures [47, 56, 57]. This study demonstrates that young Chinese men performed significantly higher than young Polish men on all three variables Internalization-General, Information and Pressures, except for Internalization-Athlete; young Chinese women performed significantly higher than young Polish women on the Information variable, but significantly lower than young Polish women on the Internalization-Athlete variable.

#### The sociocultural predictors of motivation for physical activity of young people in Poland and China

Regarding question 2, the results of this study confirm that the sociocultural attitudes towards the body in the Polish and Chinese young people predicted their physical activity objectives and the motivational function of these objectives, and there are cross-cultural differences, but this predictive function is limited to some of the variables. Importantly for this work, in the Polish and Chinese young people internalization of sociocultural standard for the body appearance, internalization of athletic body idea, and seeking information about body image was positively associated with the sense of the importance of physical activity objectives, such as Health, Company of Others, Fit, Shapely Body, Boosting Confidence, etc.; Pressures was negatively associated with the sense of the importance of physical activity objectives, such as Physical Fitness, and Pleasure from Physical Activity. This is a result that indicates that the sociocultural attitude towards the body may be of importance for Polish and Chinese young people who undertaking physical activity and how they perceive the objectives of their physical activity.

Internalization-Athlete was a positive predictor factor for the importance of physical activity objectives of Health, Fit, Shapely Body, Well-Being, Boosting Confidence, and Pleasure from Physical Activity in the Polish and Chinese young women. The self-determination theory indicated that positive health, fitness, enjoyment, mood improvement, etc. can be considered intrinsic motivation for physical activity [32, 58, 59]. Further research by the scholars showed that intrinsic motivation in young people was associated with more strenuous physical activity, and internalization of athletic body ideas was a positive predictor of them [33, 34, 60]. Mieziene et al. [60] showed that internalization of athletic body ideas leads to higher body shape requirements in young men and women, pushing them to engage in more physical activity, this supports the results of this study on the predictive effect of Internalization-Athlete on Fit, Shapely Body objective, Fulfilling the Need for Activity objective, and Promoting Physical Activity. The above research also supported the results of this study on the predictive effect of Internalization-Athlete Polish young people on Motivational Value, Time-Management, and Persistence in Action. These are important results, indicating that the internalization of the athletic body ideas promoted by the mass media may help young women in Poland and China better understand the objectives of physical activity and promote their physical activity.

Pressures were a negative predictor factor for the importance of physical activity objectives of Physical Fitness, and Pleasure from Physical Activity in the Polish and Chinese young men. The results of this study showed that the pressure young men feel about the sociocultural standards of body and appearance promoted by the mass media will affect the psychological experience of undertaking physical activities, reducing the experience of the pleasure of physical activity and the focus on physical fitness. Similar results were obtained by Anić et al. [34], showing that the perceived sociocultural pressures by young people will further change their motives for physical activity, they will exercise for reasons of body appearance rather than pleasure.

Internalization- General was a positive predictor factor for the importance of physical activity objectives of Physical Fitness and Company of Others for Polish young men, and a positive predictor factor for the importance of physical activity objectives of Escape from Everyday Life and Managing Stress for Chinese young men. Several studies support this result, stating that under the influence of the media and interpersonal interactions, young men internalize the body shape advertised by the media as their ideal body shape and in turn engage in suitable physical activities, which improve their self-esteem, stress, and social interactions [59, 61–63]. This means that the General internalization of sociocultural standards of body and appearance promoted by the mass media will contribute to the physical fitness of young Polish men through physical activity and help young Chinese men to relieve stress of life through physical activity.

Information was a significant predictor factor for the importance of physical activity objectives of Health, Fit, Shapely Body, Well-Being, Fashion, and Boosting Confidence in Polish young people, and a positive predictor factor for Time-Management. However, Information has no predictive effect on the importance of physical activity objectives and the motivational function of objectives in Chinese young men and women. A comparative study of sociocultural standards of body image for Polish and Japanese young women by Izydorczyk et al. [44] shows that Polish and Japanese young women (age 18–29) display clear cultural differences in seeking information on the sociocultural standards of body and appearance promoted by the mass media, with Polish young women responding more impulsive, such as translating information into goals for physical activity, carrying out physical activities with corresponding goals, etc., while Japanese young women choose to avoid these messages whenever possible. Polish and Japanese women grew up in completely different cultural environments. Japanese women tend to be less individualistic, which will influence them to translate the information from the mass media they acquire into individual activity, whereas Polish women who have been brought up in European culture are more willing to break with tradition and use information from the mass media to directly guide their personal physical activity [44, 54, 64, 65]. China and Japan are both parts of Asia and share a similar cultural environment. This relationship between young Poles growing up in European culture and young Chinese growing up in Asian culture is similar. The results suggest that seeking information on the sociocultural standards of body and appearance promoted by the mass media may help Polish young people to better understand the objectives of physical activity and promote their physical activity, whereas this was not shown to be the case among young Chinese.

The predictors of the motivational function of objectives showed significant differences in Polish and Chinese women and men. Such as Pressures is a positive predictor of Persistence in Action in Chinese young women, but no predictor effect in Chinese young men; conversely, Pressures is a positive predictor of Persistence in Action in Polish young men, but no predictor effect in Polish young women. Internalization-General is a negative predictor of Motivational Conflict in Polish young women, but no predictor effect in Polish young men. This may also since that young people in Poland and China grew up in very different cultural environments. The study by Wilczyńska et al. [49] indicated that Polish young people tend to respond to the influence of sociocultural attitudes toward the body promoted by the mass media through more physical activity whereas this phenomenon was close to zero in the Chinese subjects. The results prove that sociocultural attitudes toward the body promoted by mass media has a significant predictive effect on the motivational function of physical activity objectives among young Poles and tends to guide the physical activity of young Poles, whereas no such significant effect was found among young Chinese.

The analysis of the results obtained suggests that education and interventions for physical activity of young people should focus on sociocultural standards of body and appearance, influencing young people’s physical activity and associated motivation for physical activity by guiding the specific ideal body appearance promoted by the mass media.

### Limitations and future directions

This study presents a rare and unparalleled opportunity to compare and contrast the cultural differences between two distinct populations. However, the results of the study are subject to some limitations. Firstly, although the sample size was adequate for statistical analysis (total sample 467), it was relatively small for cross-cultural studies [66]. Secondly, there is a dearth of research that compares the predictive effects of different sociocultural factors on physical activity. Thirdly, it is worth noting that the study was focused on specific age groups, and there were differences in the age distributions of the samples. Future research should consider expanding similar studies to encompass different age groups, such as older adults and adolescents. Additionally, efforts should be made to increase sample sizes, ensure precise age distributions, and foster collaborative research between sports academics from both countries.

## Conclusions

This study advances the cross-cultural knowledge of how sociocultural attitudes toward the body affect motivation for physical activity among young people in Poland and China. The findings indicate that internalization of athletic and general appearance ideals, as well as perceived pressure from media and others, are universal predictors of motivation for physical activity among young people in Poland and China. However, information from media and other sources is a specific sociocultural predictor of motivation for physical activity only in Polish young people. These results imply that different cultural contexts may shape different sources of information and influence body image and motivation for physical activity. Therefore, interventions to foster physical activity among young people should take into account both universal and specific sociocultural factors that may impact their motivation.

## Data Availability

The datasets used and/or analyzed during the current study are available from the corresponding author on reasonable request.
